# Mutation of a distal gating residue modulates NADH binding in NADH:Quinone oxidoreductase from *Pseudomonas aeruginosa* PAO1

**DOI:** 10.1016/j.jbc.2023.103044

**Published:** 2023-02-18

**Authors:** Bilkis Mehrin Moni, Joanna A. Quaye, Giovanni Gadda

**Affiliations:** 1 Department of Chemistry, Georgia State University, Atlanta, Georgia, USA; 2 Department of Biology, Georgia State University, Atlanta, Georgia, USA; 3 The Center for Diagnostics and Therapeutics, Georgia State University, Atlanta, Georgia, USA

**Keywords:** NADH:quinone oxidoreductase, gating residue, binding affinity, steady-state kinetics, reductive half-reaction, hydride transfer

## Abstract

Enzymes require flexible regions to adopt multiple conformations during catalysis. The mobile regions of enzymes include gates that modulate the passage of molecules in and out of the enzyme's active site. The enzyme PA1024 from *Pseudomonas aeruginosa* PA01 is a recently discovered flavin-dependent NADH:quinone oxidoreductase (NQO, EC 1.6.5.9). Q80 in loop 3 (residues 75–86) of NQO is ∼15 Å away from the flavin and creates a gate that seals the active site through a hydrogen bond with Y261 upon NADH binding. In this study, we mutated Q80 to glycine, leucine, or glutamate to investigate the mechanistic significance of distal residue Q80 in NADH binding in the active site of NQO. The UV-visible absorption spectrum reveals that the mutation of Q80 minimally affects the protein microenvironment surrounding the flavin. The anaerobic reductive half-reaction of the NQO-mutants yields a ≥25-fold increase in the *K*_d_ value for NADH compared to the WT enzyme. However, we determined that the *k*_red_ value was similar in the Q80G, Q80L, and wildtype enzymes and only ∼25% smaller in the Q80E enzyme. Steady-state kinetics with NQO-mutants and NQO-WT at varying concentrations of NADH and 1,4-benzoquinone establish a ≤5-fold decrease in the *k*_cat_/*K*_NADH_ value. Moreover, there is no significant difference in the *k*_cat_/*K*_BQ_ (∼1 × 10^6^ M^−1^s^−1^) and *k*_cat_ (∼24 s^−1^) values in NQO-mutants and NQO-WT. These results are consistent with the distal residue Q80 being mechanistically essential for NADH binding to NQO with minimal effect on the quinone binding to the enzyme and hydride transfer from NADH to flavin.

Enzymes are very efficient catalysts for the functioning of living organisms and require some degree of flexibility for catalytic activity (1). Enzyme flexibility enables them to swap between active and inactive conformational states during a catalytic cycle. Flexible protein regions are dynamic enzyme portions that participate in a conformational switch (2, 3, 4). Gates are examples of flexible regions in enzymes (1, 3). A gate consists of individual residues, loops, secondary structural elements, or domains, switching between open and closed conformations (5). Gates control the passage of substrates, products, ions, or solvent molecules in and out of the protein (1, 6, 7, 8). The various interactions of gating residues can control the size and properties of ligands that pass through the gate. The side chain properties of gating residues dictate substrate selectivity and accessibility to the enzyme’s active site during enzyme catalysis (9). Thus, gating residues play a vital mechanistic role in controlling substrate access and product release to and from enzyme active sites by forming a path between the active site pocket and the bulk solvent (6, 7, 8, 10, 11).

The location of gating residues and the high frequency of gating residue mutations during protein functional evolution in both *Nature* and *in vitro* make them attractive protein engineering targets (1, 5, 12, 13, 14, 15). For successful protein engineering, mutation of gating residues must not be detrimental to protein function (9, 10, 11, 12, 13). Three considerations about gating residues support the idea of their attractiveness for protein engineering (1, 12, 16, 17, 18). First, the mutation of a gating residue is generally not detrimental to protein function, as gating residues are often spatially separated from the enzyme's active site. For example, the residues I135, C176, V245, and Y273 are positioned at the entrance of haloalkane dehalogenase from *Rhodococcus rhodochrous* (19). The mutants I135F, C176Y, V245F, or Y273F showed a 31-fold increase in the *k*_cat_ value with 1,2,3-trichloropropane as substrate compared to the wildtype enzyme, due to the mutant aromatic side chains’ ability to restrict water from the active site resulting in dehydration of the active site nucleophile (19). Secondly, gating residues control the opening and closing of the access pathway to and from the enzyme active site. Thus, mutations can affect ligand sizes and exchange rates, resulting in altered enzyme activity and substrate selectivity, with the replacement of bulky gating residues with smaller residues sometimes providing previously hindered bulky substrates access to the active site cavity (1, 10). For example, the D285I and D285Q mutations in toluene-4-monooxygenase from *Pseudomonas mendocina* improved the enzyme's ability to oxidize the large and bulky substrates 2-phenyl ethanol or methyl *p*-tolyl sulfide by 8- and 11-fold, respectively, and the D285S mutation improved the specific activity for styrene oxidation by 1.7-fold (1, 15). Lastly, modification of gating residues can modulate bulk solvent accessibility to the enzyme active site, affecting substrate binding and product release (20). For instance, the residue Y100 is located at the entrance of the active site in β-hydroxyacyl-acyl carrier protein dehydratase from *Helicobacter pylori* (1, 21). The mutation Y100A removes the active site gate, which increases the binding of β-hydroxy acyl-ACP to the mutant enzyme and decreases the mutant enzyme’s overall turnover due to the slow dissociation rate of the ACP product (1, 21).

The enzyme PA1024 from *Pseudomonas aeruginosa* PAO1 is a recently discovered FMN-dependent NADH:quinone oxidoreductase (NQO) (22). NQO utilizes a ping-pong bi-bi steady-state kinetic mechanism with the reduction of the enzyme-bound flavin through a hydride transfer from NADH, followed by a hydride transfer from the flavin to a quinone substrate (Fig. 1) (22, 23, 24, 25, 26, 27, 28, 29). The enzyme has a strict specificity for NADH over NADPH, unlike the eukaryotic homolog NQO1, which can use NADPH as a substrate (23, 30). Genome context analysis suggests that NQO serves a dual function in the cell by detoxifying quinones and maintaining an [NAD^+^]/[NADH] ratio favorable for fatty acid catabolism in *P. aeruginosa.* (22, 23, 24, 28, 31) NQO consists of a TIM-barrel and an extended domain, with a hinge region connecting the two domains to form the active site pocket (Fig. 2) (32). TIM-barrel domains are usually composed of eight parallel β-strands at the center of the fold and eight α-helices with βα and αβ loops connecting the secondary structures (33, 34). Extended domains, on the other hand, are formed by combining secondary structures that directly interact with the central chain atoms and typically extend from βα loops (35, 36). The βα loops of the TIM-barrel domain are located at the C-terminal ends of the β-strands, pointing toward the enzyme active site, which is crucial for the activity of TIM-barrel-containing enzymes (17, 34).

**Figure 1:**
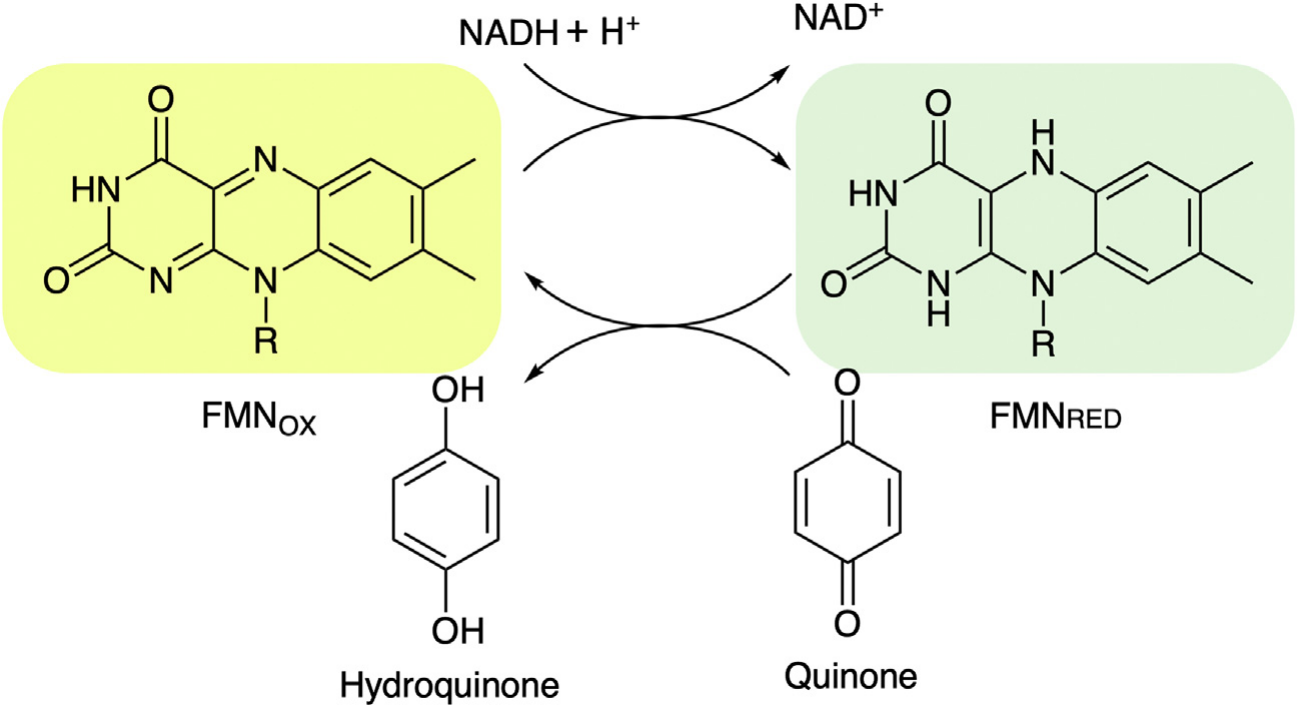
**Reaction mechanism of NQO with 1,4-benzoquinone as a substrate.** NQO, NADH:quinone oxidoreductase.

**Figure 2:**
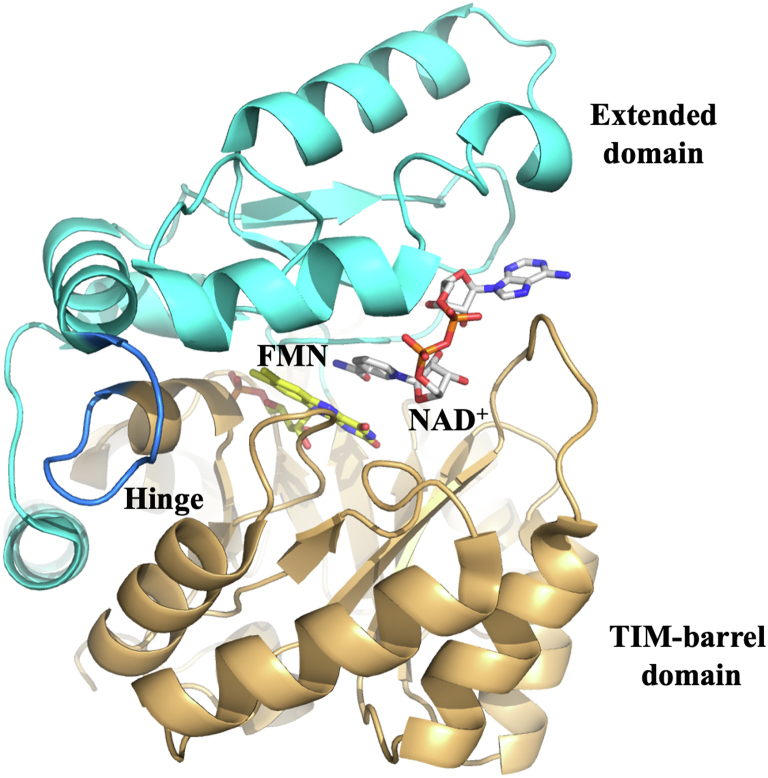
**The structure of NQO-WT with NAD**^**+**^**-bound form (2GJL)**. The NQO TIM-barrel domain is highlighted in *tan*, and the extended domain in *cyan*; the hinge region depicted in *blue* connects the TIM-barrel domain and extended domain to form the active site pocket. The *yellow* and *gray sticks* represent the FMN and NAD^+^ carbon, respectively. The PDB files were analyzed using the UCSF Chimera visualizing software (62). NQO, NADH:quinone oxidoreductase.

Previously solved crystal structures of NQO in the NAD^+^-bound and ligand-free states revealed that the βα loop 3 of the TIM-barrel domain, comprised of residues 75 to 86, moves 5.5 Å toward the active site in the presence of NAD^+^ (32). Q80 of loop 3 showed spatial variability in the ligand-free (Fig. 3*A*) and ligand-bound conformations (Fig. 3*B*) with its side chain forming a hydrogen bond (2.9 Å) with the side chain hydroxyl of Y261 in the extended domain upon NAD^+^ binding (Fig. 3*B*). In addition, the backbone amide N atom of Q80 forms hydrogen bonds with the O1 and O2 atoms of the adenine phosphate of NAD^+^ (Fig. 3*C*) (32). Based on these structural insights, loop 3 was proposed as a gate that stabilizes the enzyme-NADH complex during NQO turnover (5, 32, 37). In contrast, given the distal position of the sidechain amide N atom of Q80 from the N_5_ atom of the FMN in the active site of the enzyme (15.4 Å; Fig. 3*D*), Q80 is not expected to have a role in catalysis. However, no mechanistic or biochemical data are currently available to support the structural insight. Moreover, the structural data do not allow any sensible prediction of whether Q80 is important for quinone binding since the small substrate size compared to NAD^+^ may not require the active site gate to open to grant access to the enzyme active site.

**Figure 3:**
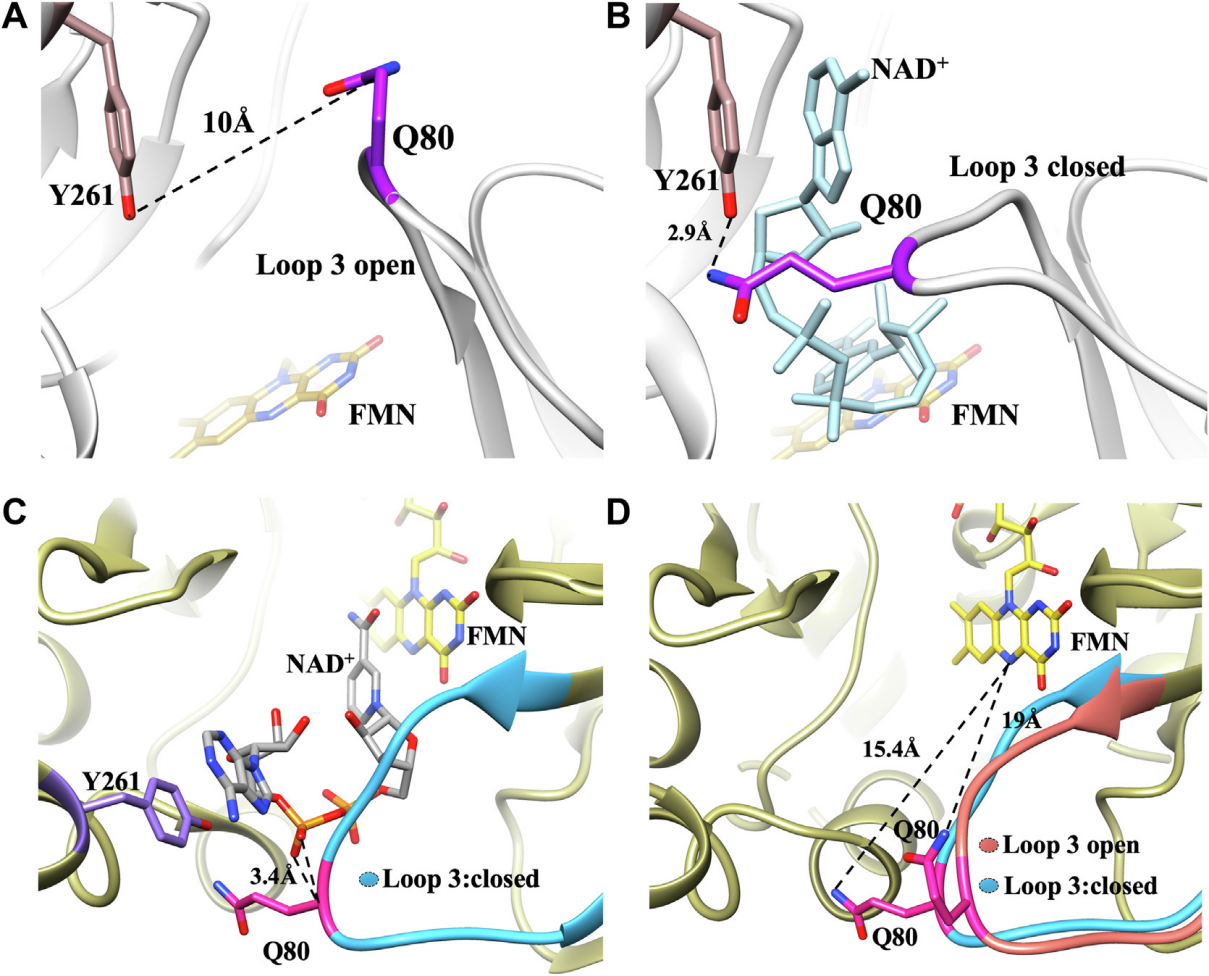
**Gating the active site in NQO-WT (PDB:****6E2A****and****2GJL****)**. *A*, active site topology of NQO showing gating residue Q80 in open conformations without ligand-bound form. *B*, gating of NQO active site by Q80 and Y261 upon NAD^+^ binding; *C*, the backbone amide N atom of Q80 establishes hydrogen bonds with the O1 and O2 atoms of the adenine phosphate of NAD^+^. *D*, distance of Q80 from the active site flavin in both open and closed conformations. In panel A and B, loop 3 is highlighted in *gray*, Q80 in *purple*, and Y261 in *brown*. The FMN and NAD^+^ are depicted in *yellow* and *cyan*, respectively. In panel C and D, loop 3 in open conformation is highlighted in *orange*, and closed conformation is highlighted in *cyan*; Q80 and Y261 are depicted in *pink* and *purple*, respectively; *yellow* and *gray sticks* represent the FMN and NAD^+^ carbon, respectively. The PDB files were analyzed using the UCSF Chimera visualizing software (62). NQO, NADH:quinone oxidoreductase.

The present study investigated the role of the distal gating residue Q80 in the binding and catalysis of NADH and quinones in NQO. Toward this end, site-directed mutagenesis, rapid kinetics, steady-state kinetics, and UV-visible absorption spectroscopy were carried out to gather mechanistic insights on the role of the distal gating residue Q80 in substrate binding and catalysis by NQO.

## Results

### Enzyme purification and UV-visible absorbance

The NQO-Q80G, Q80L, and Q80E enzymes were purified to high levels following the same protocol previously used for the wildtype enzyme (22). As for the case of the wildtype enzyme, the presence of 200 mM NaCl in a storage buffer composed of 10 mM Tris-Cl, pH 8.0, and 10% glycerol was necessary for the *in vitro* stability of purified NQO enzymes (22).

**Figure 4:**
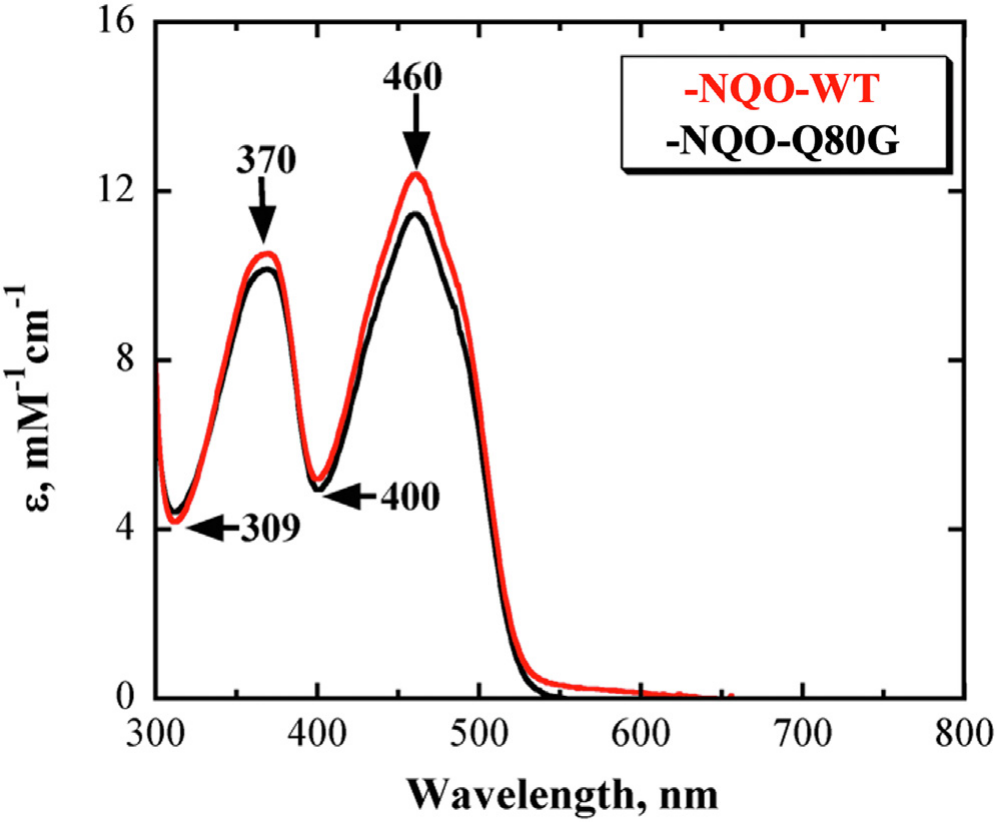
**UV-visible absorption spectra of NQO-WT (*solid red curve*) and NQO-mutant Q80G (*solid black curve*)**. The UV-visible absorption spectra were recorded in 10 mM Tris-Cl, 200 mM NaCl, and 10% v/v glycerol, at pH 8.0 and 25 °C. NQO, NADH:quinone oxidoreductase.

The spectroscopic properties of the mutant enzymes were analyzed using UV-visible absorption spectroscopy to evaluate whether the mutation of the distal residue Q80 affected the protein microenvironment surrounding the flavin in the active site of the enzyme. The UV-visible absorption spectrum of the purified mutant enzymes Q80G (Fig. 4), Q80L, and Q80E (Fig. S1) showed maximal absorbance at 370 nm and 460 nm, which is consistent with the presence of a flavin cofactor. All variant enzymes showed minimal difference in the absorption wavelength at 460 nm and 370 nm compared to the wildtype enzyme (Table 1). The FMN to protein stoichiometry was ∼0.4 for all mutant enzymes, similar to the stoichiometry determined for the wildtype enzyme (Table 1).

**Table 1:** UV-visible absorption maxima and FMN/protein stoichiometry of wildtype and mutated NQO

Enzymes	aλ_peaks_, nm	bε, mM^−1^ cm^−1^		FMN/Protein
		370	460	
WT	370, 460	10.6	12.2	0.40
Q80G	370, 460	10.0	11.2	0.35
Q80L	370, 460	10.2	11.4	0.33
Q80E	370, 460	10.5	12.5	0.41

a Spectra were recorded in 10 mM Tris-Cl, 200 mM NaCl, 10% glycerol at pH 8, 25 °C.

b Molar extinction coefficient. Standard errors of duplicates were ≤10%.

### Steady-state kinetics

**Figure 5:**
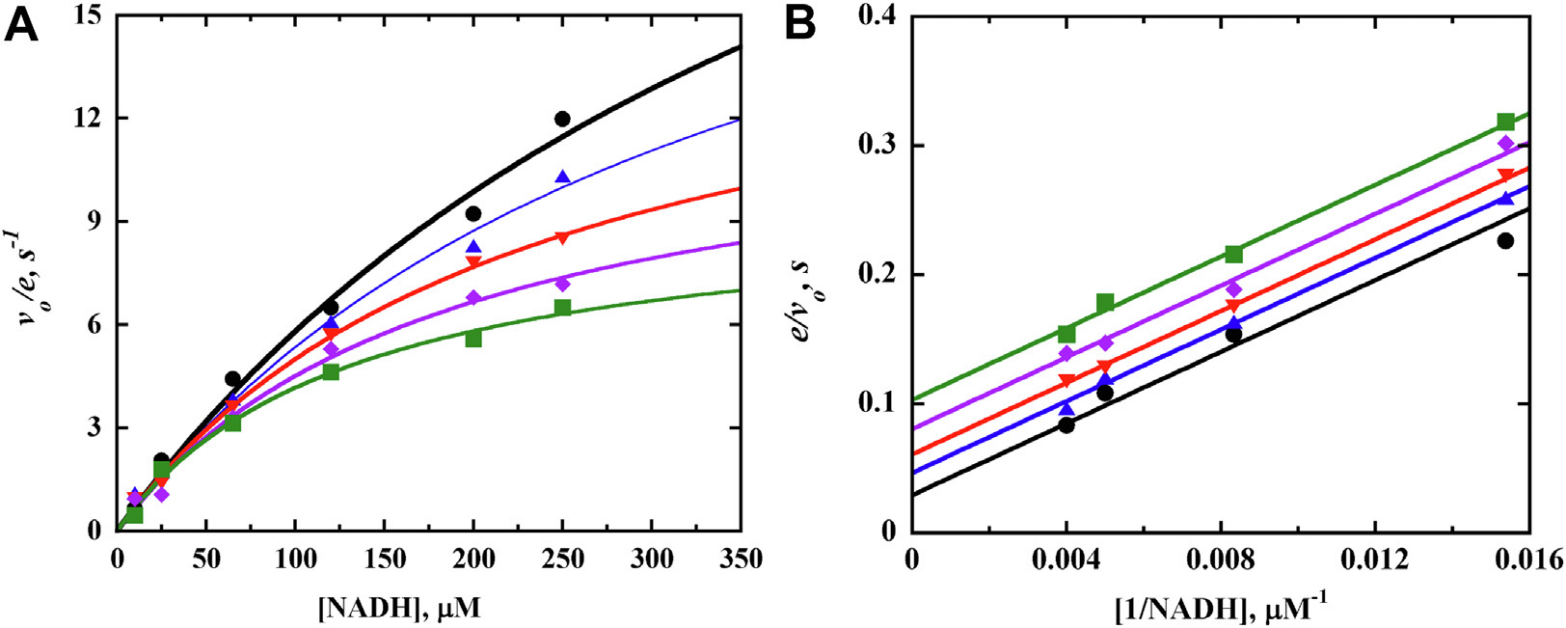
**Steady-state kinetics of NQO-Q80G with NADH and 1,4-benzoquinone as substrates**. *A*, Michaelis–Menten plot; *B*, Lineweaver–Burk plot. The initial rate of reaction was measured at varying NADH (10 μM to 250 μM) and 1,4-benzoquinone (BQ) (5 μM to 100 μM) concentrations in 20 mM KPi, 200 mM NaCl, pH 7.0, 25 °C. *Green* = 5 μM BQ, *purple* = 10 μM BQ, *red* = 25 μM BQ, *blue* = 50 μM BQ, and *black* = 100 μM BQ. Data were fitted to Equation 2. NQO, NADH:quinone oxidoreductase.

The steady-state kinetic parameters of the NQO-Q80G, Q80L, and Q80E enzymes were determined and compared to those of NQO-WT to investigate how the mutation of the gating residue Q80 affects the binding of the reducing and oxidizing substrates and catalysis of NQO. The steady-state kinetic parameters were determined by measuring the rate of NADH consumption at varying concentrations of both NADH and 1,4-benzoquinone at pH 7.0 and 25 °C. The best fits of the kinetic data of the mutant and wildtype enzymes were obtained using an equation describing a steady-state kinetic mechanism with an irreversible kinetic step between binding of the reducing substrate NADH and the oxidizing substrate 1,4-benzoquinone (Equation 2); the data are consistent with the replacement of Q80 not altering the ping-pong bi-bi mechanism of NQO (Fig. 5), which was established in a previous investigation using product inhibition studies (22). With all variant enzymes, the *k*_cat_ values differed from the wildtype NQO by less than 1.5-fold (Table 2). The *k*_cat_/*K*_NADH_ value decreased 5-fold for the Q80G enzyme and less than 2.5-fold for both Q80L and Q80E enzymes compared to the wildtype NQO. The *k*_cat_/*K*_BQ_ values for the Q80G, Q80L, and Q80E enzymes were comparable to the value determined for the NQO-WT enzyme (Table 2).

**Table 2:** aSteady-state kinetic parameters of NQO-WT, Q80G, Q80L, and Q80E with NADH and 1,4-benzoquinone as substrates

Enzyme	*k*_cat_, s^−1^	*k*_cat_/*K*_NADH,_ M^−1^s^−1^	*k*_cat_/*K*_BQ,_ M^−1^s^−1^	*K*_NADH_, μM	*K*_BQ_, μM
WT	27 ± 1	400,000 ± 30,000	930,000 ± 70,000	70 ± 10	30 ± 1
Q80G	27 ± 1	75,000 ± 8000	1,400,000 ± 150,000	360 ± 15	20 ± 1
Q80L	23 ± 1	120,000 ± 20,000	800,000 ± 80,000	180 ± 8	30 ± 1
Q80E	20 ± 1	200,000 ± 20,000	1,400,000 ± 100,000	120 ± 10	15 ± 1

a Enzymatic activity was measured by varying concentrations of both NADH and 1,4-benzoquinone in 20 mM KPi, 200 mM NaCl, pH 7.0, 25 °C. Steady-state kinetic parameters were measured following NADH oxidation using the extinction coefficient for NADH at ε_340_ = 6220 M^−1^ cm^−1^. Standard errors are from individual fits of the kinetic data.

### Reductive half-reaction with NADH

**Figure 6:**
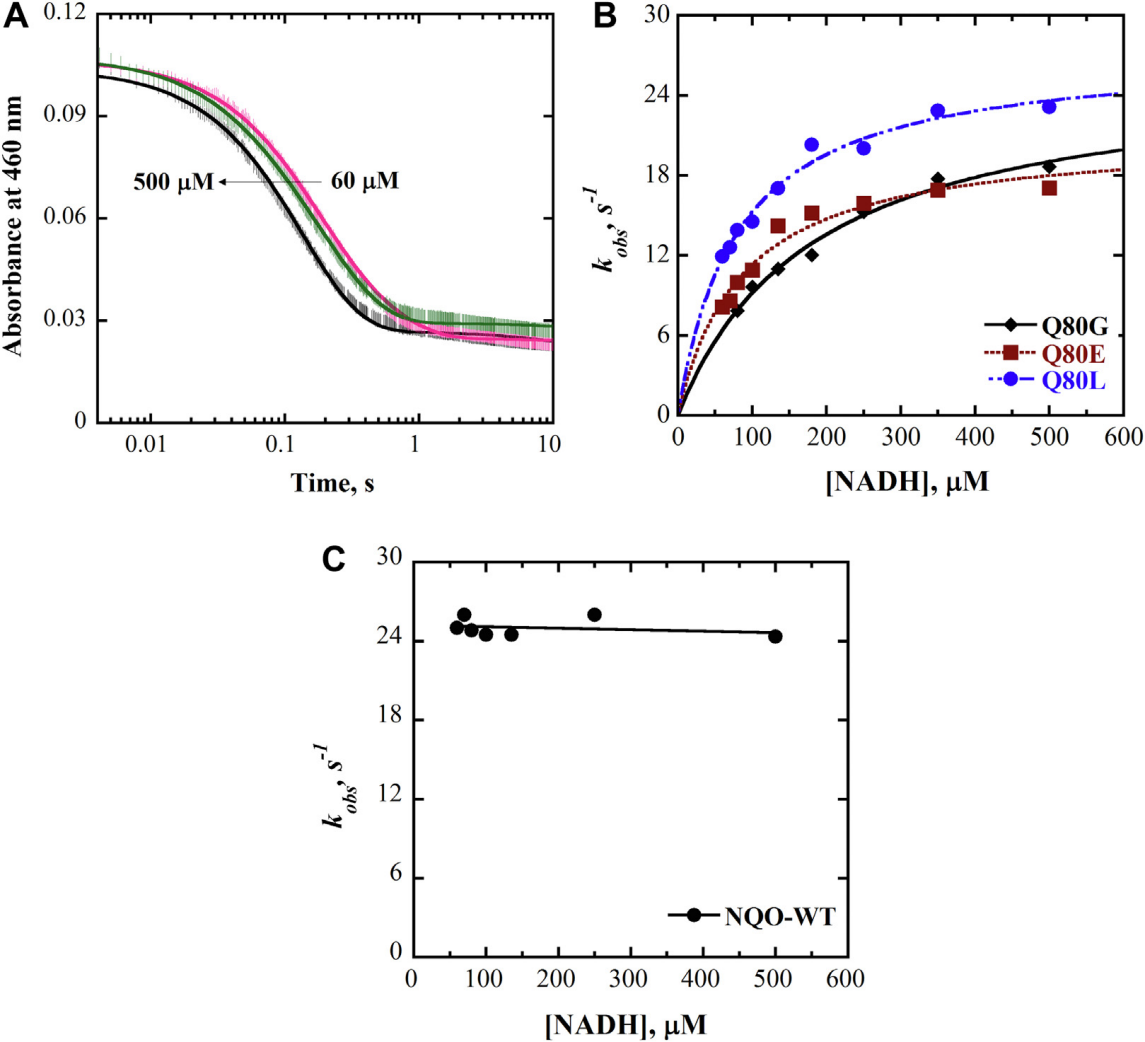
**Anaerobic reduction of NQO-Q80 mutants and WT with NADH**. *A*, stopped-flow traces of NQO-Q80G at 460 nm with varying concentrations of NADH (60–500 μM) fit Equation 3. Note the log time scale. For clarity, one out of every 10 experimental points is shown (*vertical lines*). The instrumental dead time is 2.2 ms. *B*, concentration dependence of the observed rate constant (*k*_obs_) for flavin reduction of Q80G (*black*), Q80E (*maroon*), and Q8OL (*blue*) with NADH. *C*, concentration dependence of the *k*_obs_ value for flavin reduction of NQO-WT with NADH. The *solid curve* was generated by fitting the data to Equation 4. Experiments were performed in 20 mM KPi, and 200 mM NaCl at pH 7.0 and 25 °C. NQO, NADH:quinone oxidoreductase.

The reductive-half reaction of the NQO-Q80G, Q80L, and Q80E enzymes was investigated at pH 7.0 and 25 °C by monitoring the decrease in absorbance at 460 nm upon mixing the enzyme and varying concentrations of NADH in a stopped-flow spectrophotometer anaerobically. The mutant enzymes were fully reduced with NADH in a biphasic pattern (Fig. 6*A*). The fast phase accounted for more than 95% of the total absorbance change at 460 nm and was attributed to flavin reduction. The slow phase, accounting for less than 5% of the complete change in absorbance at 460 nm, had a substrate concentration–independent *k*_obs_ value of ∼1 s^-1^, which was considerably lower than the *k*_cat_ value and was attributed to some damaged enzyme in the sample. A plot of the *k*_obs_ values with the NQO-Q80 mutant enzymes as a function of NADH concentration yielded a concentration-dependent hyperbolic curve (Fig. 6*B*) for all mutants, which allowed for the determination of the limiting rate constant for flavin reduction at saturating NADH, *k*_red_, and the dissociation constant for substrate binding, *K*_d_ (Table 3). An accurate *K*_d_ value was not determined for the wildtype enzyme as it was impossible to lower the NADH concentration below 60 μM while maintaining pseudo-first-order conditions. However, the observation that the NQO-WT enzyme was thoroughly saturated with 60 μM NADH (Fig. 6*C*) suggests a *K*_d_ value around 3 μM or less, *i.e*., at least 20-fold lower than the lowest concentration of NADH yielding full saturation of the enzyme. Thus, the *K*_d_ value for the Q80G enzyme increased by at least 60-fold, and those of the Q80L and Q80E enzymes by at least 25-fold compared to wildtype NQO (Table 3).

**Table 3:** aReductive half-reaction of NQO-WT, NQO-Q80G, NQO-Q80L, and NQO-Q80E with NADH

Enzyme	*k*_red_ (s^−1^)	*K*_d_ (μM)
WT	25 ± 1	≤ 3
Q80G	26 ± 1	183 ± 20
Q80L	27 ± 1	80 ± 5
Q80E	21 ± 1	87 ± 10

a The kinetic parameters were determined with (60–500 μM) NADH in 20 mM KPi, 200 mM NaCl, pH 7.0 at 25 °C. Standard errors are from individual fits of the kinetic data.

**Figure 7:**
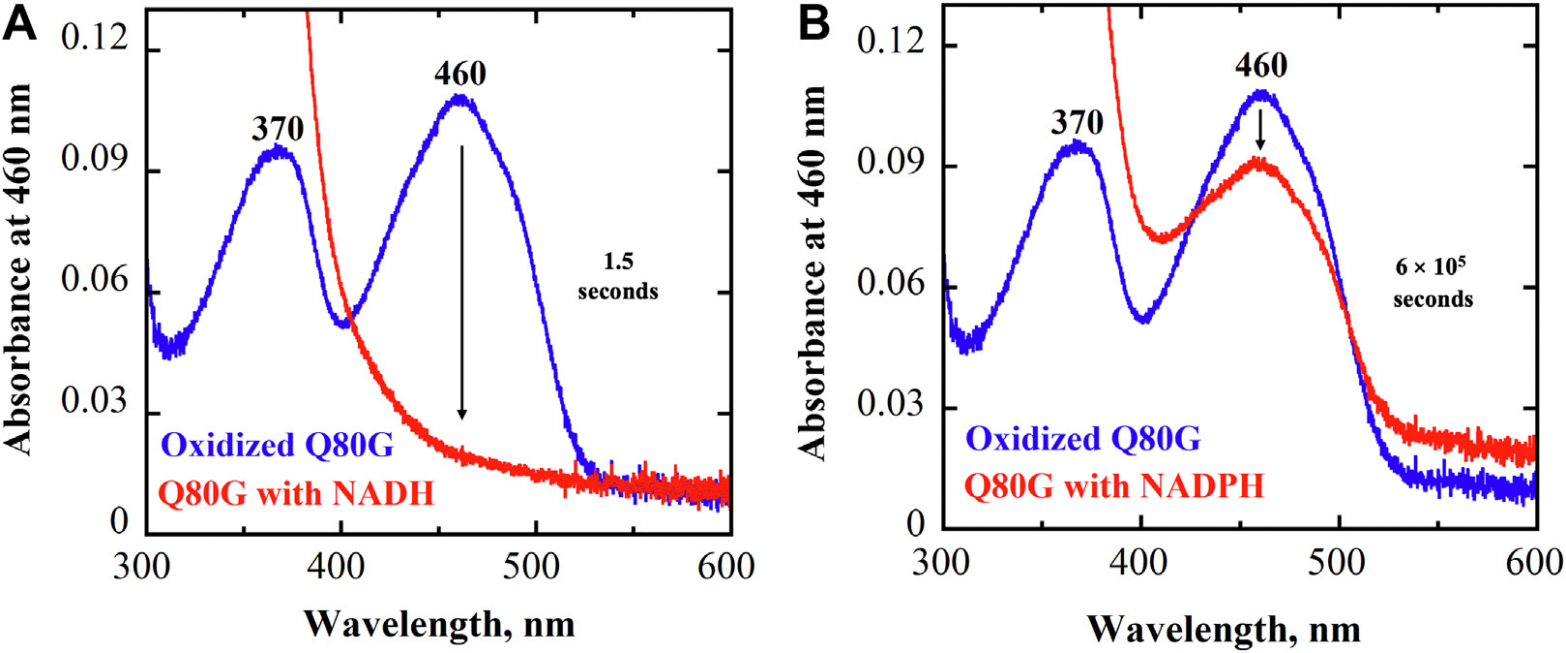
**Reduction of NQO-Q80G with NADH and NADPH**. *A* and *B*, the time-resolved absorption spectra were observed at 8 μM Q80G mixed with 60 μM NADH and 500 μM NADPH at pH 7 and 25 °C. *Blue lines* represent the spectra of oxidized flavin bound to Q80G. The *red line* of each plot corresponds to the spectrum of flavin hydroquinone recorded after 1.5 s with NADH (*A*) and 10 min with NADPH (*B*). The *arrows* represent the degree of flavin reduction, ∼90 % in A and ∼10 % in *B*. NQO, NADH:quinone oxidoreductase.

When the enzymes were anaerobically mixed with 500 μM NADPH, the enzyme-bound flavin was sluggishly reduced ∼10% over 10 min with the Q80G (Fig. 7), Q80L, Q80E, and wildtype NQO (Fig. S2). These data are consistent with NQO not acquiring the ability to use NADPH as the reducing substrate upon replacing Q80 with either glycine, leucine, or glutamate.

### *k*_cat_/*K*_m_ values with various quinones

In a previous study, NQO was shown to have broad quinone specificity (22). To evaluate whether replacing Q80 in loop 3 affected the *k*_cat_/*K*_m_ values for quinone substrates, the apparent kinetic parameters were measured at a fixed 100 μM NADH concentration using varying concentrations of 2-methyl-1,4-benzoquinone (toluquinone), 2,3-dimethoxy-5-methyl-1,4-benzoquinone, or 5-hydroxy-1,4-naphthoquinone (juglone), as substrate at pH 7.0 and 25 °C. Since NQO follows a ping-pong bi-bi steady-state kinetic mechanism, the *k*_cat_/*K*_m_ values are independent of the concentration of NADH (38). Instead, because the concentration of NADH was not saturating, both the ^app^*k*_cat_ and ^app^*K*_m_ values were apparent, not reflecting the true values, and were not used to draw any mechanistic conclusions. As summarized in Table 4, the *k*_cat_/*K*_m_ values of the NQO-Q80 mutant enzymes with all the quinone substrates tested were not significantly different from those determined with the wildtype enzyme. Thus, replacing the distal gating residue Q80 with glycine, leucine, or glutamate had a negligible impact on the rate of capture of the quinone substrate into competent enzyme-substrate complexes that proceed to catalysis.

**Table 4:** aApparent steady-state kinetics of NQO-WT, Q80G, Q80L, and Q80E enzymes with varying quinones and fixed NADH concentration

Quinones		Enzyme	^app^(*k*_cat_*/k*_m_), M^−1^s^−1^	^app^(*k*_cat_), s^−1^	^app^(*K*_m_), μM
2-methyl-1,4-benzoquinone		WT Q80G Q80L Q80E	290,000 ± 15,000	28 ± 1	96 ± 1
(Toluquinone)			300,000 ± 15,000	17 ± 1	56 ± 1
			210,000 ± 12,000	14 ± 1	68 ± 1
			350,000 ± 10,000	13 ± 1	37 ± 1
2,3-dimethoxy-5-methyl-1,4-benzoquinone		WT Q80G Q80L Q80E	300,000 ± 12,000	17 ± 1	60 ± 1
(CoQo)			280,000 ± 10,000	6 ± 1	21 ± 1
			250,000 ± 10,000	12 ± 1	48 ± 1
			400,000 ± 15,000	6 ± 1	15 ± 1
5-hydroxy-1,4-naphthoquinone		WT Q80G Q80L Q80E	720,000 ± 20,000	15 ± 1	21± 1
(Juglone)			540,000 ± 12,000	6 ± 1	10 ± 1
			820,000 ± 30,000	7 ± 1	8 ± 1
			790,000 ± 20,000	6 ± 1	8 ± 1

a The kinetic parameters were determined with 100 μM NADH in 20 mM KPi, 200 mM NaCl, pH 7.0 at 25 °C. Standard errors are from individual fits of the kinetic data.

## Discussion

In this study, the distal gating residue Q80 in βα loop 3 of *P. aeruginosa* PAO1 NQO was replaced with glycine, leucine, or glutamate to establish its role in the catalytic cycle of the enzyme. Previous studies showed that βα loop 3, comprised of residues 75 to 86, assumed a closed conformation in the crystal structure of the NAD^+^-bound NQO and an open conformation in the ligand-free enzyme (32). In the closed conformation, the side chain of Q80 interacts with the hydroxyl group of Y261 on the extended domain of the enzyme, forming a gate that closes upon the AMP tail of the enzyme-bound NAD^+^ (32). The backbone amide N atom of Q80 establishes hydrogen bonds with the O1 and O2 atoms of the adenine phosphate of NAD^+^ (32). In the open conformation, the side chain of Q80 is swung open and exposes the NAD^+^ tail to the bulk solvent. The structural data suggest that Q80 may function as a gate during the enzyme's turnover but do not provide mechanistic insights on the role of Q80 in the binding of the reducing and oxidizing substrates or the catalytic step of hydride transfer from the NADH to the flavin in the active site of the enzyme. The mutagenesis and mechanistic investigation reported here demonstrate that Q80 is important for NADH binding to NQO but not for the ensuing hydride transfer reaction from NADH to the enzyme-bound flavin or the subsequent binding of the quinone substrate to the reduced enzyme, which completes the catalytic cycle of the enzyme.

The distal gating residue Q80 is important for NADH binding in NQO. Evidence to support this conclusion comes from comparing the reductive half-reaction with NADH of the NQO mutant enzymes with Q80 substituted with glycine, leucine, or glutamate and the NQO-WT enzyme at pH 7.0 and 25 °C. The *K*_d_ value determined in the reductive half-reaction, which directly reports on the binding affinity of NQO for NADH, increased ≥25-fold in the Q80L and Q80E enzymes and ≥60-fold in the Q80G enzyme as compared to NQO-WT. The decreased affinity of the enzyme for NADH is imputable to the lack of the hydrogen bond between Y261 and the side chain of residue 80 in the mutant enzymes, as all enzymes maintain the ability to form hydrogen bonds between the backbone N atom of residue 80 and the O1 and O2 atoms of the adenine phosphate of the enzyme-bound NAD^+^. In the Q80G and the Q80L enzymes, the lack of a side chain and the nonpolar side chain prevents the formation of a hydrogen bond with Y261. In the Q80E enzyme, the electrostatic repulsion between the negatively charged carboxylate of residue 80 and the negatively charged diphosphate groups of the enzyme-bound NAD^+^ likely prevents residue 80 approach to Y261 and consequent interaction between the two residues. The effect of the lack of the Y261 hydrogen bond in the mutant enzymes is expressed minimally in the steady-state kinetic parameter *K*_m_ for NADH, which showed ≤5-fold increase for all mutant enzymes compared to the wildtype. The discrepancy in the fold increase of the *K*_d_ and *K*_m_ values likely stems from an internal isomerization of the enzyme–substrate complex, as previously established using kinetic solvent viscosity effects on the wildtype enzyme, which might have different effects on the two kinetic parameters (39). The ≥25-fold increase in the *K*_d_ value seen in the Q80L and Q80E enzymes compared to NQO-WT supports an energetic contribution of ≥8 kJ/mol estimated for the hydrogen bond between Q80 and Y261. The lack of the hydrogen bond interaction between residue 80 and Y261 in the mutant enzymes is further consistent with a destabilization of the closed conformation of the enzyme and an increased probability of βα loop 3 being in the open conformation.

Whether the replacement of Q80 results in βα loop 3 being exclusively in the open conformation or having a lower probability than in the wildtype enzyme to be in the closed conformation, Q80 is not required to capture the quinone substrates into enzyme–quinone complexes that proceed to catalysis. This conclusion is supported by the steady-state kinetic data with the mutant and wildtype enzymes turning over with NADH and quinones at pH 7.0 and 25 °C. With the NQO-Q80G, Q80L, and Q80E enzymes, the second-order rate constant *k*_cat_*/K*_m_ for 1,4-benzoquinone was not significantly different from the *k*_cat_*/K*_m_ value determined in the wildtype enzyme. Similar results were obtained when the enzyme turned over with toluquinone, Coenzyme Q_o_, or juglone instead of 1,4-benzoquinone, suggesting that the lack of an effect on the second-order rate constant for quinone capture is independent of the quinone structure. Thus, Q80’s role in substrate binding is limited to NADH, likely due to its bulky size with a ribityl-ADP tail that protrudes outside the enzyme active site, with small quinone substrates not being affected by residue 80. In the case of pyranose-2-oxidase, which oxidizes D-glucose or D-galactose and uses 1,4-benzoquinone as an alternative electron acceptor, mutations of the distal loop residue F454 decreased the *k*_cat_/*K*_m_ values for the two sugar substrates by ∼6- to 80-fold. For all mutants, there was no considerable effect on the *k*_cat_/*K*_m_ values for 1,4-benzoquinone (40). The observed disparity in the mutational effects on the *k*_cat_/*K*_m_ values of the reducing and oxidizing substrates in pyranose 2-oxidase was attributed to an open loop conformation and facile transfer of two electrons from the reduced flavin to the quinone substrate resulting in the loop being insensitive to mutagenesis during quinone reduction. Given that NQO assumes an open conformation in the absence of the NADH substrate (32), the observed effects of the Q80 mutations on NADH but not quinone binding in NQO can be explained with similar mechanistic processes as described for pyranose 2-oxidase.

The gating residue Q80 is not important for the hydride transfer from NADH to the enzyme-bound flavin. Evidence for this conclusion comes from comparing the reductive half-reaction of the Q80G, Q80L, Q80E, and NQO wildtype enzymes with NADH at pH 7.0 and 25 °C. The first-order rate constant for flavin reduction at saturating NADH concentration, *k*_red_, which reports on the hydride transfer reaction, was similar in the Q80G, Q80L, and wildtype enzymes and only ∼25% smaller in the Q80E enzyme. Considering that Q80 in βα loop 3 is ∼15 Å from the flavin N_5_ atom, its lack of participation in the hydride transfer reaction is not surprising. The results with NQO differ from those reported in a previous study of dihydrofolate reductase from *Escherichia coli*, showing that in the βF-βG loop, the replacement of G121 with valine, which is 19 Å away from the catalytic center, decreased the rate constant for hydride transfer from NADPH to the enzyme-bound 7,8-dihydrofolate by ∼200-fold (41). The G121 mutation altered the loop dynamics affecting the hydride transfer reaction in dihydrofolate reductase (41), while in NQO, irrespective of whether the loop dynamics are changed, the replacement of Q80 with G, L, and E does not affect the hydride transfer reaction. Thus, despite being part of loops that assume different conformations during enzyme turnover, G121 and Q80 play different roles in the dihydrofolate reductase and NQO, with the former exerting a long-range effect on the hydride transfer reaction and the latter devoid of such a role. The conclusions drawn in this and the previous study underscore the importance of investigating multiple enzymes and different systems to better understand the molecular mechanisms underlying long-distance effects on hydride transfer and catalysis in enzymes catalyzing similar overall reactions.

The replacement of Q80 with glycine, leucine, or glutamate does not change the rate-limiting kinetic step in the overall turnover of NQO, which is the hydride transfer from NADH to the enzyme-bound flavin. Evidence to support this conclusion comes from the steady-state kinetics and the anaerobic flavin reduction data with the Q80G, Q80L, Q80E, and wildtype enzymes at pH 7.0 and 25 °C. With all enzymes, the *k*_cat_ value was less than 15% smaller than the *k*_red_ value, consistent with the hydride transfer from NADH to the flavin being almost entirely rate-limiting for the overall enzyme turnover. A rationale that explains this observation is that irrespective of the gating residue at position 80 in NQO, the probability of βα loop 3 to stay in either the open or closed conformations do not impact the rate-limiting steps during NQO catalysis. This implies that the rate constant associated with the opening of the βα loop 3 from the closed conformation seen in the enzyme–NAD^+^ complex to the open conformation observed in the free enzyme after NAD^+^ release does not alter the rate of substrate binding or product release to the extent that would limit enzyme turnover by the mutation.

In recent years with the use of experimental and computational approaches like kinetic solvent viscosity effects (42, 43, 44) and molecular dynamics (45, 46, 47, 48) that directly report on loop mobility and flexibility, the mechanistic study of loops and their impact on enzyme function has matured and complemented structural observations of multiple enzyme states (49, 50, 51, 52, 53). This, in turn, has allowed scientists to expand the scope of mechanistic studies to incorporate dynamical effects beyond the classical structure-function paradigm that has characterized the study of enzyme behavior for decades. The study presented here demonstrates that a single point mutation of a critical residue in an enzyme loop acting as a gate that opens and closes during catalysis alters the binding affinity of the enzyme for NADH, with no effect on the binding of quinone substrates or the catalytic step of hydride transfer. The results are significant, as they demonstrate that for enzymes utilizing a Bi-Bi mechanism with two substrates, a single mutation of a residue distal from the active site, like that of Q80 in NQO, can have an impact on the binding of one substrate but not the other substrate, without affecting the catalytic step. Indeed, to our knowledge, previous studies on other enzymes with the Bi-Bi mechanism showed that mutations in gating loops affect the catalytic efficiency *k*_cat_/*K*_m_ both substrates to different extents, as described above for pyranose-2-oxidase. Additionally, the structural position of gating residues and the conformational changes induced by mutations trigger different effects on substrate binding and catalysis. A study of dihydrofolate reductase from *E.coli* in which the hairpin-forming residues M16-A19 of the active site gating loop I were replaced with a glycine resulted in decreased hydride transfer rate and the binding affinities of the enzyme for NADPH and dihydrofolate (54). Although the deletion resulted in increased substrate dissociation as reported for NADH in this study, the mutation of the gating loop I inhibited transition state stabilization, which led to a detrimental effect of the mutation on enzyme catalysis (54) as opposed to the observation made for NQO in this study.

In summary, the results presented here provide the mechanistic significance of a distal gating residue Q80 in βα loop 3 of NQO. The mutagenesis and mechanistic study demonstrate that a single point mutation of Q80 with G, L, or E can impact the binding of one substrate, NADH but not the other substrate, quinone, without affecting the catalytic step of NQO. In the future, it will be interesting to complement the mechanistic conclusions presented here with computational studies to provide a physical rationale for these conclusions.

## Experimental procedures

### Materials

The enzymes DpnI, calf intestinal alkaline phosphatase, and T4 DNA ligase were purchased from New England Biolabs; DNA polymerase (*Pfu*) was from Stratagene, and oligonucleotides were from Sigma Genosys. *E. coli* strain Rosetta(DE3)pLysS and the pET20b(+) expression vector were from Novagen. DH5α *E. coli* strain was purchased from Life Technologies, Inc.; the QIAprep spin miniprep kit, QIAquick PCR purification kit, and QIAquick, gel extraction kit were from Qiagen. HiTrap chelating HP 5-ml affinity column was from GE Healthcare, and isopropyl 1-thio-D-galactopyranoside was from Promega. All quinones were purchased from Sigma-Aldrich. NADH and NADPH disodium salts were purchased from VWR. All other reagents were of the highest purity commercially available.

### Site-directed mutagenesis and protein purification

The genes of the NQO mutant enzymes Q80G, Q80L, and Q80E were prepared using a pET20b(+) plasmid harboring the wildtype gene PA1024 as a template and mutagenic primers containing corresponding site mutations (55). The mutant genes were sent to Psomagen, Inc. for sequencing after mutagenesis. Plasmids were purified using the QIAquick spin Miniprep kit (56). The constructs containing correct mutations were transformed into chemically competent *E. coli* strain Rosetta (DE3)pLysS by heat shock for protein expression (57). The expression and purification of NQO mutant enzymes Q80G, Q80L, and Q80E followed the previously described protocol for the wildtype enzyme (22). SDS-PAGE was used to determine the purity of the enzymes (data not shown) (58).

### UV-visible absorption spectroscopy and extinction coefficient determination

An Agilent Technologies diode-array spectrophotometer model HP 8453 PC, thermostated with a water bath, was used to record the UV-visible absorbance of the enzyme-bound flavin in 10 mM Tris-Cl, 200 mM NaCl, 10% glycerol, pH 8.0, at 25 °C. The extinction coefficients of the purified NQO wildtype and mutant enzymes were determined by extracting the FMN cofactor from the enzymes using the heat denaturation method (59). The enzymes were passed through a PD-10 desalting column before heat denaturation at 100 °C for 20, 30, or 40 min (60). Denatured protein was removed by centrifugation at 20,000*g.* The concentration of the released FMN was determined spectroscopically using an ε_450_ value of 12,200 M^-1^ cm^-1^ for free FMN (22). The total protein concentration was determined using the Bradford method with bovine serum albumin as standard (61).

### Reductive half-reaction with NADH

The anaerobic reduction of the enzyme-bound flavin with NADH in 20 mM KPi, 200 mM NaCl, pH 7.0, was followed with an SF-61DX2 Hi-Tech KinetAssyst high-performance stopped-flow spectrophotometer (Bradford-on-Avon), thermostated with a water bath at 25 °C. Anaerobiosis of the instrument and all buffers, substrates, and enzyme solutions was performed according to standard procedure (22). NADH concentration was determined spectrophotometrically at 340 nm with an extinction coefficient of 6220 M^-1^ cm^-1^ (22). After mixing, the enzyme concentration was ∼6 μM, and NADH ranged from 60 to 500 μM to maintain pseudo-first-order conditions.

### *k*_cat_/*K*_m_ values with various quinones

The turnover of NQO-Q80 mutant and NQO-WT enzymes with quinones was determined at varying concentrations of toluquinone, juglone, or 2,3-dimethoxy-5-methyl-1,4-benzoquinone and fixed 100 μM NADH in 20 mM KPi, 200 mM NaCl, pH 7.0, at 25 °C. The stock solutions of quinones were prepared in 100 % ethanol. The final ethanol concentration in all reaction mixtures was kept at 1% to minimize any possible effects of this solvent on enzymatic activity. The reaction rates were measured following the NADH consumption at 340 nm, using a ε_340_ value of 6220 M^−1^ cm^−1^ (22).

### Steady-state kinetics

The steady-state kinetic parameters of the NQO-WT and NQO-mutant enzymes were determined at varying concentrations of NADH and 1,4-benzoquinone by measuring initial reaction rates for each enzyme in 20 mM KPi, 200 mM NaCl, pH 7.0, 25 °C. The concentration range for NADH was 10 to 250 μM with the Q80G and Q80E enzymes and 5 to 200 μM with both the NQO-WT and Q80L enzymes. The concentration of 1,4-benzoquinone was 10 to 200 μM with NQO-WT, 5 to 100 μM with the Q80E and Q80L enzymes, and 10 to 250 μM with the Q80G enzyme.

### Data analysis

The apparent steady-state kinetic parameters of NQO at varying concentrations of NADH and fixed concentrations of quinones were determined by fitting the initial reaction rates to the Michaelis-Menten equation (Equation 1). The steady-state kinetic parameters for the enzymatic assay were obtained by fitting the experimental points to the Michaelis-Menten equation using KaleidaGraph software (Synergy Software). The double reciprocal plot was constructed using KaleidaGraph, and global analysis was carried out using EnzFitter software (Biosoft). The initial reaction rate (v_o_/e) best fit was obtained with Equation 2, which describes a ping-pong bi-bi steady-state kinetic mechanism.

(1) $$v_{o}/e=\frac{k_{cat}\;\left[A\right]}{K_{m}+\left[A\right]}$$

(2) $$v_{o}/e=\frac{k_{cat}AB}{K_{a}B+K_{b}A+AB}$$

In the above equation, *v*_*o*_ is the initial velocity, e represents the enzyme concentration, *K*_a_ and *K*_b_ are Michaelis constants for NADH (A) and 1,4-benzoquinone (B), and *k*_cat_ is the turnover rate at saturating concentration of both substrates.

Stopped-flow traces obtained with the KinetAsyst 3 (TgK-Scientific, Bradford on-Avon) software were fit to Equation 3, which describes a double-exponential process.

(3) $$A={B_{1e^{-k}}}_{obs1^{t}}+B_{2e^{-k}obs2^{t}}+c$$

In this equation, A represents the absorbance at 460 nm at time t; B_1 and_ B_2_ mean the amplitudes of the decrease in absorbance; *k*_obs1_ and *k*_obs2_ define the observed rate constants for the change in absorbance. C is an offset value accounting for the nonzero absorbance of the enzyme-bound reduced flavin at an infinite time.

The concentration dependence for the observed rate constants of flavin reduction was analyzed with Equation 4, where S represents the concentration of NADH, *k*_red_ is the rate of flavin reduction at saturating concentration of NADH, and *K*_d_ is the dissociation constant for NADH binding.

(4) $$k_{obs}=\frac{k_{red}s}{k_{d}+s}$$

## Data availability

All data are contained within the manuscript.

## Supporting information

This article contains supporting information.

## Conflict of interest

The authors declare they have no conflicts of interest with the contents of this article.

## References

[1] Gora A., Brezovsky J., Damborsky J. (2013). Gates of enzymes. Chem. Rev..

[2] Cui Q., Karplus M. (2008). Allostery and cooperativity revisited. Protein Sci..

[3] Couture J.-F., Legrand P., Cantin L., Labrie F., Luu-The V., Breton R. (2004). Loop relaxation, A mechanism that explains the reduced specificity of rabbit 20α-hydroxysteroid dehydrogenase, A member of the aldo-keto reductase superfamily. J. Mol. Biol..

[4] Marques S.M., Daniel L., Buryska T., Prokop Z., Brezovsky J., Damborsky J. (2017). Enzyme tunnels and gates as relevant targets in drug design. Med. Res. Rev..

[5] Nestl B.M., Hauer B. (2014). Engineering of flexible loops in enzymes. ACS Catal..

[6] Nussinov R., Tsai C.-J. (2013). Allostery in disease and in drug discovery. Cell.

[7] Papaleo E., Saladino G., Lambrughi M., Lindorff-Larsen K., Gervasio F.L., Nussinov R. (2016). The role of protein loops and linkers in conformational dynamics and allostery. Chem. Rev..

[8] Pasi M., Riccardi L., Fantucci P., De Gioia L., Papaleo E. (2009). Dynamic properties of a psychrophilic α-amylase in comparison with a mesophilic homologue. J. Phys. Chem. B.

[9] Fu G., Yuan H., Li C., Lu C.-D., Gadda G., Weber I.T. (2010). Conformational changes and substrate recognition in Pseudomonas aeruginosa D-arginine dehydrogenase. Biochemistry.

[10] Richard J.P. (2019). Protein flexibility and stiffness enable efficient enzymatic catalysis. J. Am. Chem. Soc..

[11] Ouedraogo D., Souffrant M., Vasquez S., Hamelberg D., Gadda G. (2017). Importance of loop L1 dynamics for substrate capture and catalysis in Pseudomonas aeruginosa d-arginine dehydrogenase. Biochemistry.

[12] Gunasekaran K., Nussinov R. (2004). Modulating functional loop movements: the role of highly conserved residues in the correlated loop motions. ChemBioChem.

[13] Gao B., Xu T., Lin J., Zhang L., Su E., Jiang Z. (2011). Improving the catalytic activity of lipase LipK107 from Proteus sp. by site-directed mutagenesis in the lid domain based on computer simulation. J. Mol. Catal. B: Enzymatic.

[14] Coleman C.S., Stanley B.A., Pegg A.E. (1993). Effect of mutations at active site residues on the activity of ornithine decarboxylase and its inhibition by active site-directed irreversible inhibitors. J. Biol. Chem..

[15] Brouk M., Derry N.-L., Shainsky J., Zelas Z.B.-B., Boyko Y., Dabush K. (2010). The influence of key residues in the tunnel entrance and the active site on activity and selectivity of toluene-4-monooxygenase. J. Mol. Catal. B: Enzymatic.

[16] Inouye M. (2016). The first application of site-directed mutagenesis using oligonucleotides for studying the function of a protein. Gene.

[17] Thanki N., Zeelen J.P., Mathieu M., Jaenicke R., Abagyan R.A., Wierenga R.K. (1997). Protein engineering with monomeric triosephosphate isomerase (monoTIM): the modelling and structure verification of a seven-residue loop. Protein Eng. Des. Select..

[18] Yaacob N., Ahmad Kamarudin N.H., Leow A.T.C., Salleh A.B., Rahman R.N.Z.R.A., Ali M.S.M. (2019). Effects of lid 1 mutagenesis on lid displacement, catalytic performances and thermostability of cold-active Pseudomonas AMS8 lipase in toluene. Comput. Struct. Biotechnol. J..

[19] Pavlova M., Klvana M., Prokop Z., Chaloupkova R., Banas P., Otyepka M. (2009). Redesigning dehalogenase access tunnels as a strategy for degrading an anthropogenic substrate. Nat. Chem. Biol..

[20] Salvi F., Rodriguez I., Hamelberg D., Gadda G. (2016). Role of f357 as an oxygen gate in the oxidative half-reaction of choline oxidase. Biochemistry.

[21] Zhang L., Liu W., Hu T., Du L., Luo C., Chen K. (2008). Structural basis for catalytic and inhibitory mechanisms of β-hydroxyacyl-acyl carrier protein dehydratase (FabZ). J. Biol. Chem..

[22] Ball J., Salvi F., Gadda G. (2016). Functional annotation of a presumed nitronate monoxygenase reveals a new class of NADH: quinone reductases. J. Biol. Chem..

[23] Deller S., Macheroux P., Sollner S. (2008). Flavin-dependent quinone reductases. Cell Mol. Life Sci..

[24] Atia A., Alrawaiq N., Abdullah A. (2014). A review of NAD (P) H: quinone oxidoreductase 1 (NQO1); A multifunctional antioxidant enzyme. J. Appl. Pharm. Sci..

[25] Brunmark A., Cadenas E. (1989). Redox and addition chemistry of quinoid compounds and its biological implications. Free Radic. Biol. Med..

[26] Sollner S., Nebauer R., Ehammer H., Prem A., Deller S., Palfey B.A. (2007). Lot6p from Saccharomyces cerevisiae is a FMN-dependent reductase with a potential role in quinone detoxification. FEBS J..

[27] Deller S., Macheroux P., Sollner S. (2007). Flavin-dependent quinone reductases. Cell. Mol. Life Sci..

[28] Dratch B.D., Orozco-Gonzalez Y., Gadda G., Gozem S. (2021). Ionic atmosphere effect on the absorption spectrum of a flavoprotein: a reminder to consider solution ions. J. Phys. Chem. Lett..

[29] Moni B.M. (2022). Thesis, Georgia State University.

[30] Knox R.J., Jenkins T.C., Hobbs S.M., Chen S., Melton R.G., Burke P.J. (2000). Bioactivation of 5-(aziridin-1-yl)-2, 4-dinitrobenzamide (CB 1954) by human NAD (P) H quinone oxidoreductase 2: a novel co-substrate-mediated antitumor prodrug therapy. Cancer Res..

[31] Sevostyanova A., Belogurov G.A., Mooney R.A., Landick R., Artsimovitch I. (2011). The β subunit gate loop is required for RNA polymerase modification by RfaH and NusG. Mol. Cell.

[32] Ball J., Reis R.A., Agniswamy J., Weber I.T., Gadda G. (2019). Steric hindrance controls pyridine nucleotide specificity of a flavin-dependent NADH: Quinone oxidoreductase. Protein Sci..

[33] Wierenga R. (2001). The TIM-barrel fold: a versatile framework for efficient enzymes. FEBS Lett..

[34] Höcker B., Jürgens C., Wilmanns M., Sterner R. (2001). Stability, catalytic versatility and evolution of the (βα) 8-barrel fold. Curr. Opin. Biotechnol..

[35] Lockhart Z., Knipe P.C. (2018). Conformationally programmable chiral foldamers with compact and extended domains controlled by monomer structure. Angew. Chem..

[36] Van Nues R.W., Brown J.D. (2004). Saccharomyces SRP RNA secondary structures: a conserved S-domain and extended alu-domain. Rna.

[37] Afonine P.V., Grosse-Kunstleve R.W., Echols N., Headd J.J., Moriarty N.W., Mustyakimov M. (2012). Towards automated crystallographic structure refinement with phenix.refine. Acta Crystallogr. D Biol. Crystallogr..

[38] Mitchell D.A., Krieger N. (2022). Looking through a new lens: expressing the Ping Pong bi bi equation in terms of specificity constants. Biochem. Eng. J..

[39] Quaye J.A., Ball J., Gadda G. (2022). Kinetic solvent viscosity effects uncover an internal isomerization of the enzyme-substrate complex in Pseudomonas aeruginosa PAO1 NADH: quinone oxidoreductase. Arch. Biochem. Biophys..

[40] Spadiut O., Tan T.C., Pisanelli I., Haltrich D., Divne C. (2010). Importance of the gating segment in the substrate-recognition loop of pyranose 2-oxidase. FEBS J..

[41] Cameron C.E., Benkovic S.J. (1997). Evidence for a functional role of the dynamics of glycine-121 of Escherichia coli dihydrofolate reductase obtained from kinetic analysis of a site-directed mutant. Biochemistry.

[42] Feng C., Kedia R.V., Hazzard J.T., Hurley J.K., Tollin G., Enemark J.H. (2002). Effect of solution viscosity on intramolecular electron transfer in sulfite oxidase. Biochemistry.

[43] Grant B.D., Hemmer W., Tsigelny I., Adams J.A., Taylor S.S. (1998). Kinetic analyses of mutations in the glycine-rich loop of cAMP-dependent protein kinase. Biochemistry.

[44] Gadda G., Sobrado P. (2018). Kinetic solvent viscosity effects as probes for studying the mechanisms of enzyme action. Biochemistry.

[45] Williams J.K., Wang B., Sam A., Hoop C.L., Case D.A., Baum J. (2022). Molecular dynamics analysis of a flexible loop at the binding interface of the SARS-CoV-2 spike protein receptor-binding domain. Proteins: Struct. Funct. Bioinform..

[46] Park H., Yeom M.S., Lee S. (2004). Loop flexibility and solvent dynamics as determinants for the selective inhibition of cyclin-dependent kinase 4: comparative molecular dynamics simulation studies of CDK2 and CDK4. ChemBioChem.

[47] Olufsen M., Smalås A.O., Moe E., Brandsdal B.O. (2005). Increased flexibility as a strategy for cold adaptation: a comparative molecular dynamics study of cold-and warm-active uracil DNA glycosylase. J. Biol. Chem..

[48] Raghav P.K., Verma Y.K., Gangenahalli G.U. (2012). Molecular dynamics simulations of the Bcl-2 protein to predict the structure of its unordered flexible loop domain. J. Mol. Model..

[49] Hanson J.A., Duderstadt K., Watkins L.P., Bhattacharyya S., Brokaw J., Chu J.-W. (2007). Illuminating the mechanistic roles of enzyme conformational dynamics. Proc. Natl. Acad. Sci. U. S. A..

[50] Repka L.M., Chekan J.R., Nair S.K., Van Der Donk W.A. (2017). Mechanistic understanding of lanthipeptide biosynthetic enzymes. Chem. Rev..

[51] Palzkill T. (2018). Structural and mechanistic basis for extended-spectrum drug-resistance mutations in altering the specificity of TEM, CTX-M, and KPC β-lactamases. Front. Mol. Biosci..

[52] Fry D.C., Kuby S.A., Mildvan A.S. (1986). ATP-Binding site of adenylate kinase: mechanistic implications of its homology with ras-encoded p21, F1-ATPase, and other nucleotide-binding proteins. Proc. Natl. Acad. Sci. U. S. A..

[53] Schnell J.R., Dyson H.J., Wright P.E. (2004). Structure, dynamics, and catalytic function of dihydrofolate reductase. Annu. Rev. Biophys. Biomol. Struct..

[54] Li L., Wright P.E., Benkovic S.J., Falzone C.J. (1992). Functional role of a mobile loop of Escherichia coli dihydrofolate reductase in transition-state stabilization. Biochemistry.

[55] Steffens D.L., Williams J.G. (2007). Efficient site-directed saturation mutagenesis using degenerate oligonucleotides. J. Biomol. Tech. JBT.

[56] Pronobis M.I., Deuitch N., Peifer M. (2016). The miraprep: a protocol that uses a miniprep kit and provides maxiprep yields. PLoS One.

[57] Inoue H., Nojima H., Okayama H. (1990). High efficiency transformation of Escherichia coli with plasmids. Gene.

[58] Fujita T., Shiota K., Yoshikawa J., Ogawa S., Aoyagi H. (2019). Simple method for analyzing the purity of protease-containing samples by acid-treatment SDS-PAGE. J. Biosci. Bioeng..

[59] Hayes W.A., Mills D.S., Neville R.F., Kiddie J., Collins L.M. (2011). Determination of the molar extinction coefficient for the ferric reducing/antioxidant power assay. Anal. Biochem..

[60] Kim H.-S., Huber K.C. (2007). Simple purification (desalting) procedure to facilitate structural analysis of an alkali-solubilized/neutralized starch solution by intermediate-pressure size-exclusion chromatography. J. Agric. Food Chem..

[61] Kruger N.J., Walker J.M. (2009). The Protein Protocols Handbook. Springer Protocols Handbooks.

[62] Pettersen E.F., Goddard T.D., Huang C.C., Couch G.S., Greenblatt D.M., Meng E.C. (2004). UCSF Chimera—a visualization system for exploratory research and analysis. J. Comput. Chem..

