# Supplementary material for: Mutation of a distal gating residue modulates NADH binding in NADH:Quinone oxidoreductase from Pseudomonas aeruginosa PAO1
Source: J Biol Chem. 2023 Feb 18;299(4):103044. doi: 10.1016/j.jbc.2023.103044 (PMC10033279; doi:10.1016/j.jbc.2023.103044)
Supplement: Supplemental data [file mmc1.docx]

**Mutation of a Distal Gating Residue Modulates NADH Binding in NADH:Quinone Oxidoreductase from *Pseudomonas aeruginosa* PAO1**

Bilkis Mehrin Moni^1^, Joanna A. Quaye^1^, and Giovanni Gadda^1,2,3,*^

^1^Departments of Chemistry, ^2^Biology, and ^3^The Center for Diagnostics and Therapeutics, Georgia State University, Atlanta, GA 30302-3965


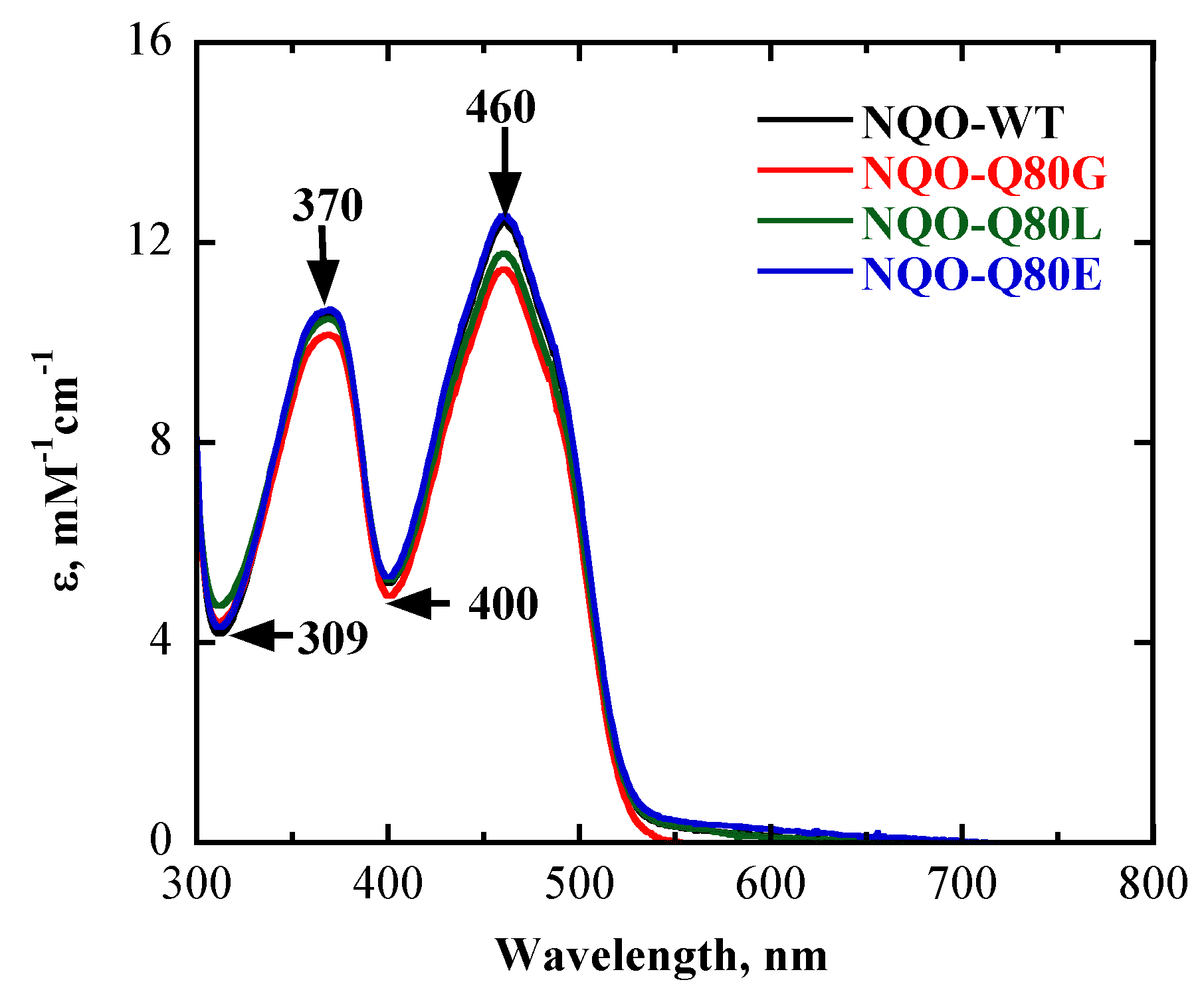


**Figure S1: UV-visible absorption spectra of NQO-WT (solid black curve) and NQO-mutant Q80G (solid red curve), Q80L (solid green curve), and Q80E ( solid blue curve).** The UV-visible absorption spectra were recorded in 10 mM Tris-Cl, 200 mM NaCl, and 10% v/v glycerol, at pH 8.0 and 25 ^o^C.

**
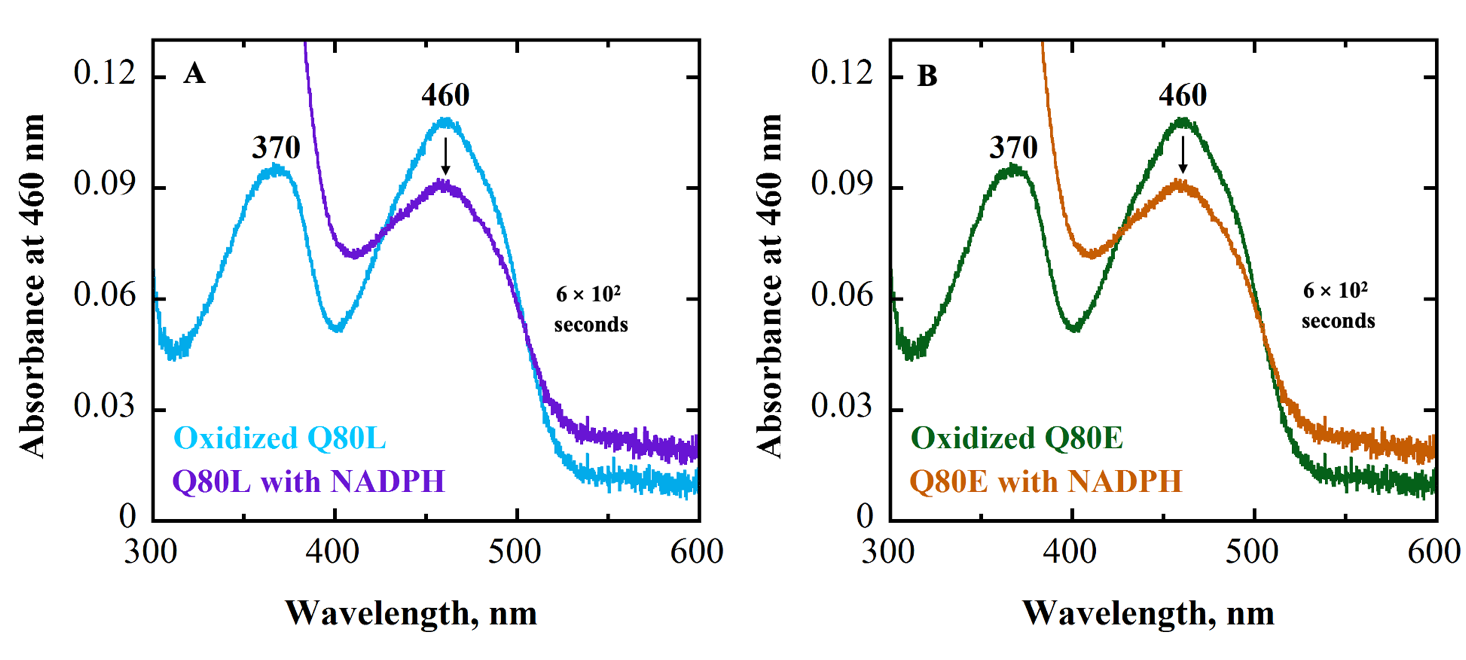
**

**Figure S2:** **Reduction of NQO-Q80L and NQO-Q80E with NADPH (A) & (B).** The time-resolved absorption spectra were observed at 8 µM Q80L and Q80E mixed with 500 µM NADPH at pH 7 and 25 ^o^C. In panel (A) & (B) Cyan and green lines represent the spectra of oxidized flavin bound to Q80L and Q80E, respectively. The purple and orange lines correspond to the spectrum of flavin hydroquinone recorded after 10 mins with NADPH in Q80L and Q80E, respectively. The arrows represent the degree of flavin reduction, ~10 % in A and B.
